# P-1522. Transcriptional Cascade in Cytomegalovirus Infection

**DOI:** 10.1093/ofid/ofaf695.1706

**Published:** 2026-01-11

**Authors:** Arya Zandvakili, Ming Li, Qiaolin Hu, Mrutyunjaya Parida, Jeffery Meier

**Affiliations:** University of Iowa, Iowa City, IA; University of Iowa, Iowa City, IA; University of Iowa, Iowa City, IA; University of Iowa, Iowa City, IA; Iowa City Veterans Affairs Healthcare System, Iowa City, Iowa

## Abstract

**Background:**

During lytic infection, cytomegalovirus (CMV), like other Herpesviruses, follows a cascade of gene expression that is classically modeled by categorizing genes as Immediate Early (IE, involved in triggering Early gene expression), Early (E, involved in viral DNA replication), and Late (L, involved in virion assembly). Historically, this cascade is characterized by measuring levels of mRNA and protein products of viral genes. However, mRNA and protein levels are a function of multiple different processes. Without isolating a specific step in gene expression, identifying mechanisms that coordinate the expression of IE, E, and L genes is challenging. Here we aim to characterize the cascade of gene expression specifically at the level of transcription, the process by which DNA is expressed as RNA. By isolating transcriptional activity, we may be able to better identify the mechanisms that coordinate expression, which may serve as therapeutic targets.

**Methods:**

We conducted a novel analysis of a previously published Precision Run-On Sequencing (PRO-seq) dataset. PRO-seq allows for genome-wide measurement of transcriptional activity at single-nucleotide resolution. This dataset was produced by infecting cultured human foreskin fibroblasts (HFFs) with CMV and performing PRO-seq at 4-, 12-, 24-, 48-, and 72-hours post-infection (covering the entire CMV life cycle).

**Results:**

Hierarchical clustering was used to cluster both coding and non-coding genes into groups with similar temporal patterns of transcription. Some groups of genes are transcriptionally active at defined time-periods, consistent with the classic cascade model. Additionally, we find evidence of genes with transcriptional activity inconsistent with the classic cascade model – specifically, genes with transcriptional activity persistent throughout viral life cycle and genes with intermittent transcriptional activity.

**Conclusion:**

Preliminary analysis of CMV transcription activity suggests coordinated transcription as well as inconsistencies with the classic cascade model of Herpesvirus gene expression. Next steps include: (1) test results through separate assays of transcriptional activity; (2) identify mechanisms of coordinated transcription; and (3) assess cell-type differences.

**Disclosures:**

All Authors: No reported disclosures

